# Methylglyoxal induces oxidative stress and ferroptosis of renal tubular epithelial cells in acute and chronic kidney injury mice

**DOI:** 10.3389/fcell.2025.1604575

**Published:** 2025-09-19

**Authors:** Yongzheng Zhang, Xuwu Zhang, Jiayu Ren, Hao Sun, Zhe Yang, Jianning Wang

**Affiliations:** ^1^ Department of Urology, The First Affiliated Hospital of Shandong First Medical University & Shandong Provincial Qianfoshan Hospital, Jinan, China; ^2^ Medical Science and Technology Innovation Center, Shandong First Medical University & Shandong Academy of Medical Sciences, Jinan, China; ^3^ School of Public Health, Shandong First Medical University & Shandong Academy of Medical Sciences, Jinan, China; ^4^ School of Preventive Medicine Sciences (Institute of Radiation Medicine), Shandong First Medical University & Shandong Academy of Medical Sciences, Jinan, China

**Keywords:** methylglyoxal, kidney injury, ferroptosis, reactive oxygen species, GSH, selenoprotein

## Abstract

Methylglyoxal (MG), which is a highly reactive dicarbonyl compound, has been increasingly recognized due to its role in the pathogenesis of kidney injury, particularly in diseases such as diabetic nephropathy and hypertensive nephropathy. MG promotes advanced glycation end product (AGE) formation and directly impairs cell function. MG levels are significantly increased in various renal diseases. This study aimed to investigate the relationship between MG and ferroptosis, which is a form of cell death involving iron-dependent lipid peroxidation (LPO), during kidney injury. Animal and cellular experiments were conducted to verify that ferroptosis occurred after MG treatment, and RNA sequencing (RNA-Seq) was performed to analyse changes in gene expression profiles after MG exposure. A mouse model that mimicked human kidney injury was established by administering folic acid, and the MG and D-lactate levels in serum and tissues were measured. *In vitro* experiments involved treating human HK-2 renal tubular epithelial cells (TECs) with MG and assessing markers of cytotoxicity, oxidative stress, and ferroptosis. MG levels were significantly increased in serum and renal tissues from patients with renal injury and from model mice. In HK-2 cells, MG treatment induced cytotoxicity, increased intracellular reactive oxygen species (ROS) levels, triggered ferroptosis by increasing LPO, and decreased glutathione (GSH) levels. These findings suggest a potential association between MG accumulation and kidney injury and indicate that MG promotes ferroptosis specifically in renal TECs. This study provides new insights into the molecular mechanisms underlying MG-induced kidney injury and potential directions for developing new therapeutic strategies.

## Introduction

Acute kidney injury (AKI) and chronic kidney disease (CKD) are interrelated conditions that have significant health implications worldwide. AKI, which is characterized by a sudden decrease in renal function, is associated with high morbidity and mortality rates, and it is increasingly being recognized as a risk factor for the development of CKD and end-stage renal disease (ESRD) (Bucaloiu et al., 2012; Coca et al., 2012; Hoste et al., 2018). CKD, on the other hand, is a progressive condition that often arises from healing responses to AKI, and it is characterized by the gradual loss of kidney function over time (Chawla et al., 2014; Venkatachalam et al., 2015). The transition from AKI to CKD is complex and involves multiple mechanisms, including inflammation, fibrosis, and metabolic reprogramming within renal tubular epithelial cells (TECs) (Guzzi et al., 2019; Gao et al., 2020) Understanding this transition is crucial, as it not only affects patient outcomes but also contributes to the increasing burden of CKD on healthcare systems worldwide (See et al., 2019; Kurzhagen et al., 2020). The occurrence of kidney injury, whether AKI or CKD, leads to metabolic reprogramming in renal TECs, which is characterized by an abnormal increase in glycolysis.

Methylglyoxal (MG) is a highly reactive dicarbonyl compound that is primarily produced as a byproduct of glycolysis, and its levels increase under conditions of high blood glucose, such as in diabetes (Nigro et al., 2017). The pathological state of kidney injury is characterized by an aberrant increase in glycolysis, resulting in a concomitant increase in MG levels. MG is also generated through other pathways, including ketone body metabolism, aminoacetone oxidation, and lipid peroxidation (LPO) (Phillips and Thornalley, 1993). The glyoxalase system includes glyoxalase 1 (Glo1), glyoxalase 2 (Glo2), and glutathione (GSH). The glyoxalase pathway is a biochemical process by which MG is converted to D-lactate and GSH through the intermediary S-D-lactoylglutathione by Glo1 and Glo2 (Christopher et al., 1995; Thornalley, 1990); this pathway is the main pathway for MG detoxification, and the level of D-lactate that is produced is related to MG production (Lin et al., 2011; Huang et al., 2013; Yu et al., 2016; Lin et al., 2021), with Glo1 functioning as the rate-limiting enzyme (Shaheen et al., 2014). An imbalance between MG production and Glo1 activity can lead to MG accumulation, which has been implicated in renal injury. Moreover, MG contributes to diabetic nephropathy, potentially through increased glomerular protein modification and oxidative stress (Giacco et al., 2014). The regulation of Glo1 activity, either by its inhibition or overexpression, can mimic or prevent the impact of the diabetic state on kidney function, respectively (Brouwers et al., 2011). MG primarily reacts with proteins via arginine (Arg) residues, resulting in the formation of isomeric cyclic hydroimidazolones (MG-Hs) and, occasionally, other products, such as Arg-Lys dimers. Of all the MG-protein adducts, the MG-H1 isomer accounts for approximately 90% (Thornalley et al., 2003). These covalent adducts that are formed by MG are collectively called advanced glycation end products (AGEs) (Rabbani and Thornalley, 2015). The accumulation of MG and its derived AGEs has been implicated in various pathological conditions, including renal damage. In the context of kidney injury, MG can induce oxidative stress, inflammation, DNA damage (Kong et al., 2024), and apoptosis in renal cells (Rabbani and Thornalley, 2018). Furthermore, MG has been shown to activate stress-related signalling pathways, such as the p38MAPK, JNK, and NF-κB signalling pathways, and to increase the expression of RAGE, which can further exacerbate inflammation and oxidative stress (Brouwers et al., 2014). These mechanisms underlying cell death, including apoptosis and potentially other forms, such as ferroptosis, are critical for understanding the role of MG in renal injury.

Ferroptosis is an iron-dependent form of regulated cell death that has been implicated in the pathogenesis of AKI and CKD (Friedmann Angeli et al., 2014). The precise reasons why ferroptosis occurs in renal injury are not fully understood, but recent studies suggest that MG may play a key role in inducing ferroptosis (Linkermann et al., 2014a; Stockwell et al., 2017). This novel cell death pathway involves LPO, and it is distinct from other forms of cell death, such as apoptosis and necrosis (Dixon et al., 2012). Ferroptosis plays a significant role in the context of kidney injury, particularly in diseases such as diabetic nephropathy and hypertensive nephropathy (Rabbani and Thornalley, 2018). Studies have demonstrated that the levels of characteristic ferroptosis markers, such as 4-hydroxynonenal (4-HNE) and malondialdehyde (MDA), are increased in kidney tissues that are affected by injury (Linkermann et al., 2014b). The mechanism underlying ferroptosis is complex and includes factors that are related to iron metabolism, LPO, and antioxidative stress mechanisms (Stockwell et al., 2017). The interplay between ferroptosis and renal injury is further supported by evidence showing that ferroptosis can be induced by conditions that lead to oxidative stress, such as ischaemia‒reperfusion injury, which is a common cause of AKI (Linkermann et al., 2014b). The potential role of MG in ferroptosis highlights the need for further research to understand the interplay between MG and renal injury; such research could provide new therapeutic targets for kidney diseases.

This study aimed to investigate the relationship between MG-induced kidney injury and ferroptosis by conducting animal experiments and cell experiments to verify that ferroptosis occurs after MG treatment. Additionally, RNA sequencing (RNA-Seq) was performed to analyse changes in gene expression profiles after MG exposure to further elucidate the molecular mechanisms involved in MG-induced ferroptosis. These findings may provide new insights into the mechanism underlying kidney injury while also identifying potential molecular targets for the development of strategies to treat this condition.

## Methods

### Materials

The Creatinine Assay Kit (cat. no. C011-2-1), Urea Assay Kit (cat. no.C013-2-1), GSH (cat. no.A006-2-1) and MDA (cat. no.A003-4-1) Assay Kit were purchased from Nanjing Jiancheng Bioengineering Institute. A D-LA (cat. no.BC5350) and Hexokinase Activity Assay Kit (cat. no.BC0745) was purchased from Beijing Solaibao Technology Co., Ltd. The MG Assay Kit (cat. no.JL-T0942-96) was purchased from Shanghai Jianglai Biotechnology Co., Ltd. The Annexin V-FITC/PI (cat. no. 40302ES50) cell apoptosis detection kit was purchased from Shanghai Yisheng Biotechnology Co., Ltd. C11-BODIPY (cat. no.D3861) was purchased from Thermo Fisher Scientific. DCFH-DA (cat. no.HY-126793), RhoNox-1 (cat. no.HY-D1533), semarbazide hydrochloride (cat. no.HY-Y0470), L-Glutathione reduced (cat. no.HY-D0187R), glyoxalase I inhibitor (cat. no.HY-15167A), folic acid (cat. no.HY-16637) and MG (cat. no. HY-W020014) were purchased from MedChemExpress. All the chemicals were used as received without further purification.

### Mouse model

All animal experiments and protocols were approved by the Animal Ethics Committee at the First Affiliated Hospital of Shandong First Medical University (2024022101). A total of 15 male C57BL/6J mice (6–8 weeks old; body weight 20–24 g) were purchased from Beijing Vital River Laboratory Animal Technology Co., Ltd. (Beijing, China). The mice were housed under specific pathogen-free (SPF) conditions at 22°C ± 2°C with 50%–60% humidity, a 12-hour light/dark cycle, and *ad libitum* access to standard rodent chow and water. For invasive procedures, mice were anesthetized using 3% isoflurane (RWD Life Science, China) in oxygen (1 L/min) via a precision vaporizer, followed by maintenance at 1.5%–2% isoflurane. At the study endpoint, mice were euthanized by cervical dislocation under deep anesthesia (5% isoflurane), followed by bilateral thoracotomy to confirm death. Death was verified by absence of spontaneous breathing, heartbeat, and corneal reflex. All procedures followed the guidelines of the Institutional Animal Care and Use Committee. The study duration was 15 days, with acute and chronic kidney injury phases monitored at 3 and 15 days post-folic acid (FA) injection, respectively. Control mice received intraperitoneal injections of 0.3% sodium bicarbonate (vehicle) in parallel with experimental groups at Day 0, and were euthanized at matched timepoints (3 and 15 days). Folic acid was dissolved in a solution of 0.3 mmol/L sodium bicarbonate and intraperitoneally administered at a dose of 250 mg/kg body weight (Yan, 2021). Kidney injury was confirmed by measuring blood urea nitrogen (BUN) levels using a commercial assay kit. Successful model establishment was defined as a BUN increase >2-fold compared to baseline levels.

### Cell culture and cytotoxicity determination

HK-2 cells purchased from the Wuhan Procell Life Technology Co., LTD were cultured in Dulbecco’s modified eagle medium (DMEM) with glucose at a concentration of 4.5 g/L (Gibco, USA), supplemented with 10% fetal bovine serum (Sijiqing, China) and 100 U/mL penicillin-streptomycin (Gibco, USA) according to the handling procedure (Zhou, 2020). The cells were cultured at 37 °C with 5% CO_2_ in a humidified incubator (ThermoFisher Scientific, USA). For cell counting, HK-2 cells were seeded into 6-well plates (Nest, China) at a density of 5 × 10^5^ cells/well and incubate overnight, HK-2 cells were counted using a Cell drop instrument (DeNoVix, USA). For cell death assessment, HK-2 cells were inoculated into 96-well plates (Nest, China) with a density of 8 × 10^3^ cells per well and incubated for 24 h. Then the culture was continued for 24 h with 100 μL of Dulbecco’s Modified Eagle medium replacement medium containing the Methylglyoxal in the concentration range of 0 ∼ 1 mM. The cells were washed twice with sterile PBS. 100 μL of fresh DMEM medium and 10 μL of CCK-8 solution (Beyotime, China) were added and incubated at 37 °C for 1 h. Finally, cell viability was quantified by measuring the absorbance at 450 nm with Microplate Reader (ThermoFisher Scientific, USA).

### Quantitative real-time PCR (RT-qPCR)

Total RNAs were isolated from tissues or cells using Trizol reagent (Thermo Fisher Scientific, USA). For cDNA synthesis, 1 μg of total RNA was reverse-transcribed using the MonScript™ RTIII All-in-One Mix (Monad, China) in a 20 μL reaction system containing 4 μL of RTIII Mix and 1 μL of dsDNase. The reaction conditions were as follows: 37°C for 2 min (genomic DNA digestion), 5°C for 15 min (cDNA synthesis), and 85°C for 5 min (enzyme inactivation). The resulting cDNA was stored at −20 °C or used immediately for qPCR.

Real-time qPCR was performed using GoTaq qPCR Master Mix (Accurate Biology, China) on a CFX Connect qPCR instrument (Bio-Rad, USA) Each 20 μL reaction contained 10 μL of 2× SYBR Premix Ex Taq™, 0.8 μL each of forward and reverse primers (10 μM; sequences listed in Table 1), and 2 μL of cDNA template. All samples were run in triplicate on 96-well plates using the QuantStudio 6 Flex system. The amplification protocol included an initial denaturation at 95 °C for 30 s, followed by 40 cycles of 95 °C for 5 s (denaturation) and 60 °C for 30 s (annealing/extension). Relative mRNA expression levels were calculated using the ΔΔCt method, with β-actin as the internal reference gene.

**TABLE 1:** Relevant primer sequences.

Gene	Species	Forward primer	Reverse primer
GLO1	Mouse	TGGATTTGGTCACATTGGGATTGC	GGTCTTGAATGAACGCCAGTCC
GLO2	Mouse	GGCTGAAGGTTTATGGAGGTGATG	CGAAGTATGGCAGGGTGTTGAC
HK2	Mouse	TGATCGCCTGCTTATTCACGG	AACCGCCTAGAAATCTCCAGA
LDHD	Mouse	GTGTCAACCTCCTGATGCTGTG	CGGTGCCTGTGCCAAATGG
KIM	Mouse	CCAAGAAGACCCACAACTACAAGG	GTTGGAGGAGTGGAGGTAGAGAC
GPX4	Mouse	ATAAGAACGGCTGCGTGGTGAAG	TAGAGATAGCACGGCAGGTCCTTC
SLC7A11	Mouse	ACCACCATCAGTGCGGAGGAG	ATGGAGCCGAAGCAGGAGAGG
TFR1	Mouse	GGCTCTGGCTCTCACACTCTC	GGCATTTGCGACTCCCTGAATAG
FTH1	Mouse	TGCCAAATACTTTCTCCACCAATCTC	CCCGCTCTCCCAGTCATCAC
PTGS2	Mouse	GTGCCTGGTCTGATGATGTATGC	TGAGTCTGCTGGTTTGGAATAGTTG
ACSL4	Mouse	GCGTTCCTCCAAGTAGACCAACC	ACGTTCACACTGGCCTGTCATTC
β-ACTIN	Mouse	GGCTGTATTCCCCTCCATCG	CCAGTTGGTAACAATGCCATGT
GLO1	Human	TTCGGTCATATTGGAATTGCTGTTC	GCCATCAGGATCTTGAATAAATGCC
GLO2	Human	AGATCACTCACCTGTCCACACTG	CGGGCTTGCTCACGAAGTAAC
HK2	Human	CGTGCCCGCCAGAAGACATTAG	CTTGCTCAGACCTCGCTCCATTTC
LDHD	Human	TGATGACGCCGAGGAACTGG	GCTGCCGCTTGCCCATTC
GPX4	Human	CCCGATACGCTGAGTGTGGTTTG	TCTTCGTTACTCCCTGGCTCCTG
SLC7A11	Human	ACGGTGGTGTGTTTGCTGTCTC	GCTGGTAGAGGAGTGTGCTTGC
TFR1	Human	CGGCAAGTAGATGGCGATAACAG	CACAGCAATAGTCCCATAGCAGATAC
FTH1	Human	TGAGCAGGTGAAAGCCATCAAAG	GAGATATTCCGCCAAGCCAGATTC
PTGS2	Human	TCCACCAACTTACAATGCTGACTATG	ATCATCAGGCACAGGAGGAAGG
ACSL4	Human	GCTCTGTCACACACTTCGACTCAC	TTCCCTGGTCCCAAGGCTGTC
β-ACTIN	Human	GATCATTGCTCCTCCTGAGC	ACTCCTGCTTGCTGATCCAC

### Western blotting

The total protein was extracted from HK-2 cells treated with methylglyoxal, hydrogen peroxide, and LPS using RIPA lysis buffer (Beyotime, China) supplemented with 1% phenylmethylsulfonyl fluoride (Beyotime, China). The protein concentration in each group was determined and adjusted using the BCA protein assay (Thermo Scientific, USA). Next, denatured protein samples (15 μg per lane, diluted in 5× loading buffer and boiled at 95 °C for 5 min) were separated on 10% SDS-PAGE gels and transferred to PVDF membranes (Millipore, USA) using a Pyxis Gel Processor (China). The PVDF membranes were incubated with 5% skim milk (Solarbio Life Science, China) in TBST for 1 h at room temperature. Blocked PVDF membranes were then incubated with the following primary antibodies in the primary antibody dilution buffer (Beyotime, China): anti-GPX4 rabbit IgG (1:2000 dilutions, cat. no.67763-1-lg, proteintech, China), anti-SLC7A11 rabbit IgG (1:1000 dilutions, cat. no.A13685, ABclonal, China), anti-GLO1 rabbit IgG (1:1000 dilutions, cat. no.15140-1-AP, proteintech, China), anti-SEPHS2 rabbit IgG (1:1000 dilutions, cat. no.14109-1-AP, Abclonal, China) and anti-β-actin mouse IgG (1:20000, cat. no.66009-1-lg, Proteintech, China). β-actin was used as an internal reference to determine the loading weights of the proteins. After incubation with primary antibodies overnight at 4 °C, the membranes were sufficiently washed with TBST and incubated with goat anti-rabbit or rabbit anti-goat IgG (1:5000 dilutions, cat. no. SA00001-2 Proteintech, China) secondary antibody conjugated with HRP (1:5000 dilutions, cat. no.SA00001-1 Proteintech, China) for 1 h. Sufficiently washed membranes were imaged with a freshly prepared ECL hypersensitive chemiluminescence solution on the ChemiDoc XRS+ gel imaging system (Bio-Rad, USA).

### Flow cytometry

HK-2 cells were treated with 600 μM MG for 24 h in 12-well plates (Nest, China), cellular LPO was observed. Cells that were treated with 100 μM cumene hydroperoxide (CH) for 2 h served as the positive control group. The cells were subsequently treated with trypsin and subjected to centrifugation at 450 × g for 5 min to collect the cells against the inner wall of the tube. The, the treated cells were washed twice with warm PBS and stained with C11-BODIPY for 30 min at 37 °C, following the manufacturer’s instructions. The labelled cells were subsequently washed again with warm PBS. The emission peaks of the oxidized and reduced forms of C11-BODIPY were then detected by flow cytometry (AttuneTM NxT, Thermo Fisher Scientific, USA) at 488 nm and 561 nm.

HK-2 cells were treated with 600 μM MG for 24 h in 12-well plates (Nest, China), and cellular reactive oxygen species (ROS) levels were observed. Cells that were treated with 200 μM hydroperoxide for 2 h served as the positive control group. The cells were subsequently treated with trypsin and subjected to centrifugation at 450 × g for 5 min to collect the cells against the inner wall of the tube. The treated cells were then washed twice with warm PBS and stained with 10 μM DCFH-DA for 30 min at 37 °C, following the manufacturer’s instructions. The labelled cells were subsequently washed again with warm PBS. The emission peaks of the oxidized and reduced forms of DCFH-DA were then detected by flow cytometry (AttuneTM NxT, Thermo Fisher Scientific, USA) at 488 nm.

HK-2 cells were seeded in 12-well plates (Nest, China), and viability was assessed after a 24-hour treatment with 600 μM MG, preceded by pretreatment with Ferrostatin-1(Fer-1), Z-VAD-FMK(Z-VAD), or 3-Methyladenine (3-MA). Cells that were treated with absolute ethanol served as the positive control group. The cells were subsequently treated with trypsin and subjected to centrifugation at 450 × g for 5 min to collect the cells against the inner wall of the tube. The treated cells were washed twice with hot PBS and stained with 5 μM propidium iodide (MCE, China) for 30 min on ice, following the manufacturer’s instructions. The emission peaks of the oxidized and reduced forms of propidium iodide were then detected by flow cytometry (AttuneTM NxT, Thermo Fisher Scientific, USA) at 545 nm.

All flow cytometry data were analyzed using FlowJo software (v10.8.1; FlowJo LLC, OR, USA).

### Histological and cellular imaging

To systematically evaluate the pathological role of methylglyoxal (MG) in kidney injury, we employed a combined histopathological and cellular imaging strategy. Kidney tissues from mice were fixed in 4% paraformaldehyde, paraffin-embedded, and sectioned at 4 μm thickness. Sections underwent deparaffinization in xylene, rehydration through graded ethanol series, and heat-induced antigen retrieval in citrate buffer (pH 6.0, 95 °C, 10 min), followed by blocking with 3% bovine serum albumin (BSA) for 30 min at room temperature. For immunohistochemical (IHC) staining, sections were incubated overnight at 4 °C with primary antibodies against 4-HNE (1:50, cat. no. MA5-27570, Thermo Fisher Scientific, USA) or MG-H1 (1:50, cat. no.STA-011, Cell Biolabs, USA), followed by application of biotinylated secondary antibodies (1:200, cat. no.GB23303, Servicebio, China) and streptavidin-HRP complexes. Diaminobenzidine (DAB, Servicebio, China) was used for chromogenic development with hematoxylin counterstaining, and slides were digitally scanned using an Olympus VS200 whole-slide scanner (×20 objective). For immunofluorescence (IF) analysis under identical primary antibody conditions, Fluor 488-conjugated secondary antibodies (Fluor 488-conjugated anti-rabbit IgG, cat. no.111-585-003, Jackson, USA) were applied for 1 h at room temperature, with nuclear counterstaining achieved using DAPI-containing mounting medium (Servicebio, China), and images captured via Olympus BX53 fluorescence microscope (40× oil immersion lens). Parallel cellular-level investigations utilized HK-2 cells plated in glass-bottom dishes (2 × 10^5^ cells/dish, Nest). For lipid peroxidation assessment, cells were stained with 5 μM C11-BODIPY for 30 min, while intracellular Fe^2+^ and ROS were detected using 10 μM RhoNox-1 and 10 μM DCFH-DA, respectively. Nuclear visualization was achieved through Hoechst 33342 staining (Invitrogen, USA), with all confocal imaging performed on a Nikon A1R HD25 system using sequential laser excitation (405 nm, 488 nm, 561 nm). Quantitative analysis of IHC/IF signals was conducted using ImageJ (NIH) to calculate integrated density values from 10 random fields per sample, while confocal data quantification (C11-BODIPY oxidation ratios, RhoNox-1/DCFH-DA intensities) utilized NIS-Elements AR 5.02 software.

### MG measurement

Kidney tissues were homogenized in PBS to prepare 10% kidney homogenates. The MG levels of the kidney homogenates were determined according to the instructions provided with the reagent kits. The values were standardized to the total weights of the kidney tissues. To visualize cellular MG, HK-2 cells were plated in 6-well plates (Nest, China) at a density of 5 × 10^5^ cells/well and incubated overnight at 37 °C. The cells were subsequently exposed to 300 μM hydrogen peroxide (H_2_O_2_) for 24 h at 37 °C. The MG levels of the cells were determined according to the instructions provided with the reagent kits. The values were standardized according to the total HK-2 cell counts.

### RNA-seq

RNA integrity was assessed using the Bioanalyzer 2100 system (Agilent Technologies, CA, USA). Library preparation for transcriptome sequencing included the preparation of both non-strand-specific and strand-specific libraries. Messenger RNA was purified from total RNA using poly-T oligo-bound magnetic beads. After fragmentation, first-strand cDNA was synthesized using random hexamer primers, and second-strand cDNA synthesis was subsequently performed. For strand-specific libraries, dUTP was used instead of dTTP. Libraries were prepared after end repair, A-tailing, adapter ligation, size selection, amplification, and purification. These libraries were quantified with a Qubit instrument and real-time PCR, and their size distributions were assessed with a bioanalyzer. HK-2 cells were treated with 600 μM MG for 24 h, with three replicates per treatment group. Moreover, a control (Ctrl) group, including three replicates of HK-2 cells without MG treatment, was also included. This setup allowed the assessment of MG-induced effects on gene expression. Clustering and sequencing were conducted on the Illumina platform, employing the “Sequencing by Synthesis” method. Fluorescently labelled dNTPs, DNA polymerase, and adapter primers were added to the sequencing flow cell for amplification. As each sequencing cluster extended its complementary strand, the addition of each fluorescently labelled dNTP released a corresponding fluorescence signal. The sequencer captured these signals and converted them into sequencing peaks through computer software; thus, the sequence information of the target fragment was obtained.

### Statistical analysis

Data are expressed as mean ± standard deviation (SD). The significance between two groups was evaluated using Student’s t-test. One-way analysis of variance (ANOVA) was employed for comparisons among multiple groups. A p-value less than 0.05 was considered statistically significant. Following ANOVA significance (p < 0.05), Tukey’s honestly significant difference (HSD) *post hoc* tests were applied for all pairwise comparisons. This method controls the family-wise error rate at α = 0.05 for multiple comparisons, as recommendedfor experiments with balanced group sizes. (*p < 0.05, **p < 0.01, ***p < 0.001, ****p < 0.0001).

## Results

### The MG concentration is elevated during renal injury

**FIGURE 1:**
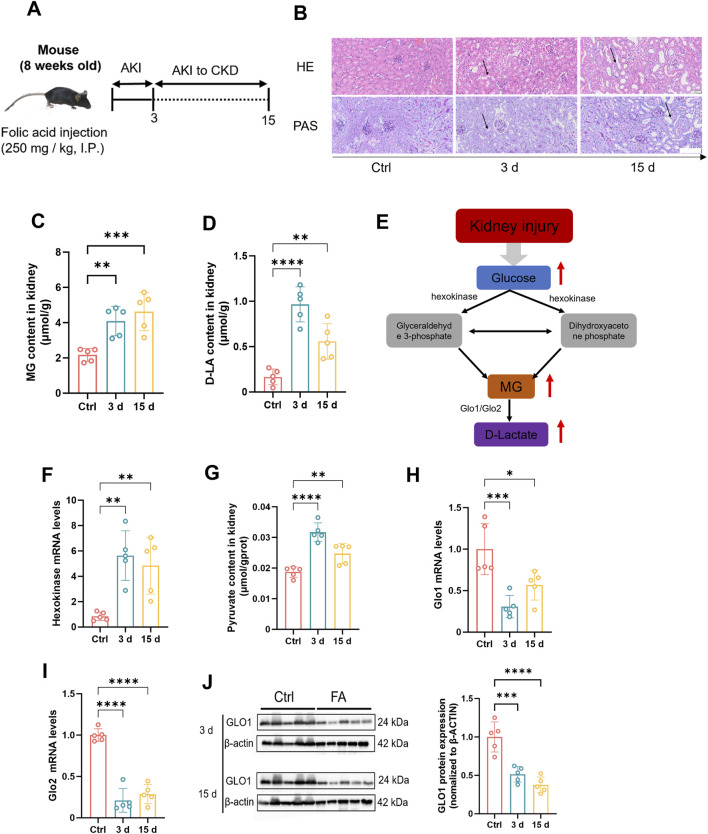
The concentration of methylglyoxal is elevated during renal injury. **(A)** Folic acid (250 mg/kg body weight) was injected intraperitoneally into the mouse model. This figure was designed by the authors. **(B)** Mouse kidney tissues were stained with HE (n = 5) and PAS (n = 5). Scale bar: 20 μm. The content of **(C)** MG and **(D)** D-lactate in kidney tissue of mice (n = 5). **(E)** Metabolic pathways underlying MG creation and clearance. **(F)** Gene expression levels of Hexokinase 2 in mouse kidney tissue (n = 5). **(G)** Pyruvate content in kidney tissue of mice (n = 5). Gene expression levels of **(H)** Glo1 and **(I)** Glo2 in mouse kidney tissues (n = 5). **(J)** GLO1 protein expression level and quantitative analysis in kidney tissue of mice (n = 5). ****P < 0.0001, ***P < 0.001, **P < 0.01, *P < 0.05.

First, we induced a mouse kidney injury model by intraperitoneal injection of folic acid (250 mg/kg in 0.3% sodium bicarbonate solution). The experimental mice were divided into acute and chronic groups to simulate different stages of kidney injury (Figure 1A). The results demonstrated significant morphological disparities in the kidneys of mice across various stages of renal injury. Specifically, during the acute phase, mice exhibited signs of congestion and edema, whereas during the chronic phase, they exhibited atrophy (Supplementary Figure S1A). Furthermore, notable differences in the weights of both kidneys were observed (Supplementary Figure S1B). Histopathological analysis revealed vacuolar degeneration in proximal tubular epithelial cells, accompanied by brush border shedding and tubular lumen obstruction, were observed in both the acute and chronic injury groups, with chronic injury exhibiting significantly greater severity compared to acute injury (Figure 1B). Moreover, there was a significant increase in the serum creatinine levels (Supplementary Figure S1C), urea nitrogen levels (Supplementary Figure S1D), and N-acetyl glucosidase activity in mouse kidney tissues (Supplementary Figure S1E), confirming the successful establishment of the kidney injury model. On the basis of these findings, MG and D-lactate levels in mouse kidney tissues were measured (Figures 1C,D). Compared with those in normal mice, MG and D-lactate concentrations were markedly greater with kidney injury, which was consistent with the MG and D-lactate levels that were observed in the serum samples (Supplementary Figures S2A,B). These results further suggest a strong correlation between MG and kidney injury. Furthermore, on the basis of previous studies (Luengo et al., 2019), it has been established that MG is derived primarily from the glycolytic pathway (Figure 1E). Therefore, we investigated the genetic activity of hexokinase in mouse kidney tissues (Figure 1F). In cases of kidney injury, we observed an increase in hexokinase activity as well as pyruvate content in renal tissues (Figure 1G), which, combined with our previous and current findings, suggests that the increased activity of the glycolytic pathway plays a significant role in the increased production of MG during tissue injury. Additionally, we examined the gene expression levels of three key enzymes that are involved in MG metabolism—Glo1 (Figure 1H), Glo2 (Figure 1I), and D-lactate dehydrogenase (Supplementary Figure S2C)—along with the protein levels of GlO1 (Figure 1J). The results indicated a decrease in the activities of enzymes that are associated with these pathways in mice with kidney injury. This suggests that the metabolic pathway of MG is inhibited during tissue injury, establishing conditions that promote its accumulation. Consequently, we hypothesize that increased glycolytic pathway activity contributes to increased MG production under pathological conditions, such as kidney injury, and that inhibition of the MG degradation pathway after injury may be a crucial factor that contributes to its accumulation within kidney tissues.

### Ferroptosis is observed in mice with kidney injury, concomitant with an increase in MG levels

**FIGURE 2:**
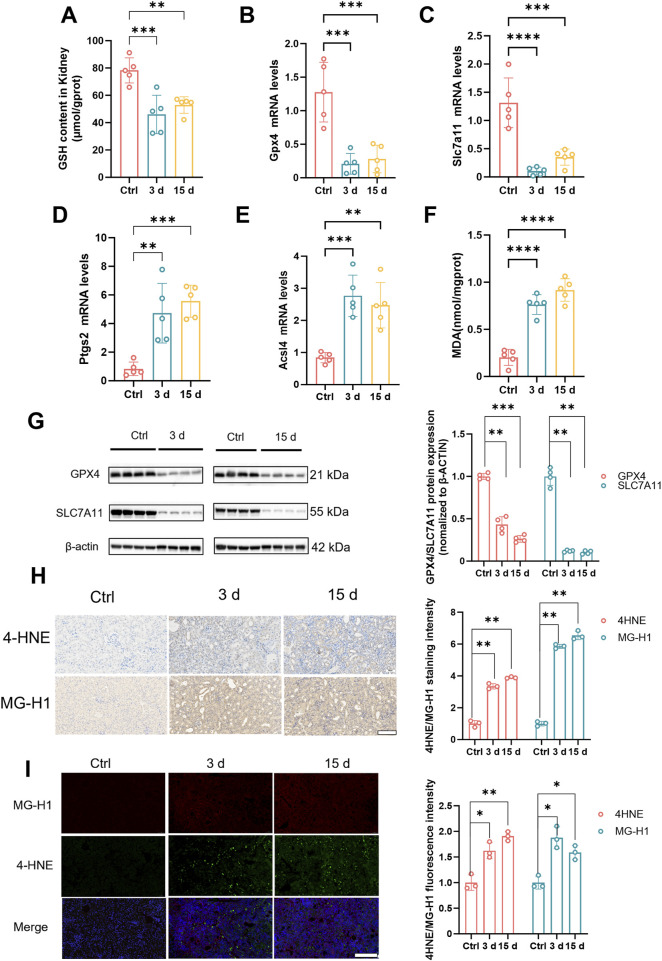
The impact of methylglyoxal on renal tubular epithelial cells. **(A)** Glutathione content in kidney tissues of mice at different stages (n = 5). **(B–E)** Gene expression levels of Gpx4, Slc7a11, Ptgs2 and Acsl4 in kidney tissues of mice at different stages (n = 5). **(F)** MDA content in kidney tissue of mice at different stages (n = 5). **(G)** The protein expression levels of GPX4 and SLC7A11 in kidney tissues of mice in different stages (left panel) and correspondence quantitative statistical analysis (right panel) (n = 4). **(H)** Immunohistochemistry images of 4HNE and MG-H1 in kidney tissues of mice at different stages (left panel) and correspondence quantitative analysis of the images (right panel). Scale bar: 50 μm (n = 3). **(I)** Immunofluorescence images of 4HNE and MG-H1 in kidney tissues of mice at different stages (left panel) and the quantitative analysis of the images were performed (right panel). Scale bar: 20 μm. (n = 3). ****P < 0.0001, ***P < 0.001, **P < 0.01, *P < 0.05.

A significant elevation in MG levels was observed in both patients and mouse models during the pathological state of kidney injury. To investigate this phenomenon thoroughly, we quantified the GSH contents of mouse kidney tissues at various time points (Figure 2A). The results revealed a significant reduction in the GSH contents in the kidney tissues of mice with kidney injury compared with those in normal kidney tissues, indicating a potential disruption of antioxidant defence mechanisms and ferroptosis within the kidneys. Numerous previous studies have established a close association between renal injury and the occurrence of ferroptosis. Therefore, we further examined the expression levels of genes and proteins that are associated with GSH biosynthesis, including Glutathione Peroxidase 4 (Gpx4) and Solute Carrier Family 7 Member 11 (Slc7a11). Our findings revealed that both the gene and protein expression levels of these two markers were significantly downregulated in kidney tissues that were affected by kidney injury (Figures 2B,C,G). In addition, we investigated other hallmark genes associated with ferroptosis, including Prostaglandin endoperoxide Synthase 2 (Ptgs2)、Acyl-CoA Synthetase Long-Chain Family Member 4 (Acsl4), Ferritin Heavy Chain 1(Fth1) and Transferrin receptor 1 (Tfr1). The expression levels of these genes were significantly elevated in the kidney tissues of mice with renal injury (Figures 2D,E; Supplementary Figures S3A,B). Concurrently, MDA contents were increased at different stages of kidney injury in mice (Figure 2F), providing further evidence for the occurrence of ferroptosis in renal tissues. To confirm the occurrence of ferroptosis, renal tissue sections were stained at various time points to detect the 4HNE levels, which is an end product associated with ferroptosis. Immunohistochemistry and immunofluorescence staining revealed significant upregulation of the level of 4HNE staining in the kidney tissues of mice with renal injury (Figure 2H). Additionally, a similar trend was observed for the level of 4HNE staining, which binds to MG (Figure 2I). These findings suggest that the accumulation of MG may be closely associated with the occurrence and progression of ferroptosis, potentially even promoting ferroptosis. Collectively, our results reveal characteristic changes that are indicative of renal ferroptosis in mice with renal injury and suggest the potential involvement of MG in this process. The concurrent elevation of MG levels and ferroptosis markers in injured kidneys suggests potential mechanistic interplay, though causation remains to be established.

### Cellular injury results in increased intracellular MG levels and triggers ferroptosis

**FIGURE 3:**
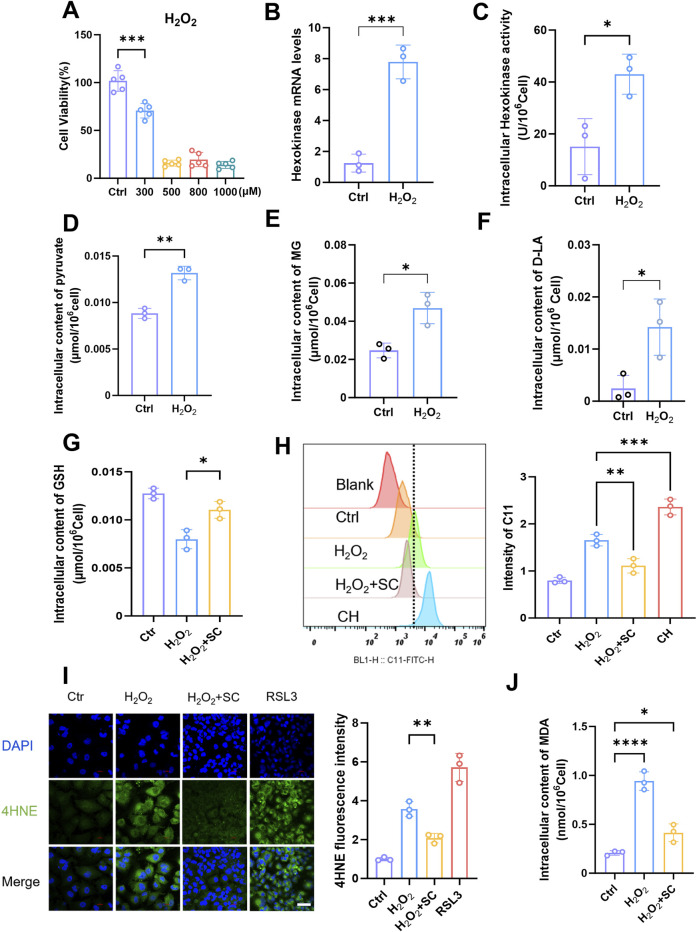
Elevation of methylglyoxal in kidney injury is accompanied by ferroptosis of renal tubular epithelial cells. **(A)** The cell viability of HK-2 cells was assessed after 24 h stimulation with varying concentrations of H_2_O_2_ (n = 5). **(B)** The relative expression of hexokinase gene in HK-2 cells after stimulated with H_2_O_2_ (300 μM) for 24 h (n = 3). **(C)** Intracellular hexokinase activity in HK-2 cells after stimulated with H_2_O_2_ (300 μM) for 24 h (n = 3). **(D–F)** The content of intracellular pyruvate, MG, and D-lactate in HK-2 cells after stimulated with H_2_O_2_ (300 μM) for 24 h (n = 3). **(G)** The intracellular GSH content (n = 3) was measured after stimulating HK-2 cells with H_2_O_2_ (300 μM) for 24 h and subsequently adding SC (1 mM). **(H)** Flow cytometry analysis the degree of lipid peroxidation in HK-2 cells after stimulated with H_2_O_2_ (300 μM) and then treated with SC (1 mM) (left panel), and quantified by Flow JO (right panel) (n = 3).**(I)** After 24 h of stimulation with H_2_O_2_ (300 μM) and also treated with SC (1 mM), HK-2 cells were subjected to immunofluorescence detection of 4HNE expression (left panel), RAS-selective lethal 3 (RSL3) serves as the positive control, followed by quantitative analysis (right panel. Scale bar: 20 μm (n = 3). **(J)** Intracellular MDA content of HK-2 cells after stimulated with H_2_O_2_ (300 μM) for 24 h (n = 3). ****P < 0.0001, ***P < 0.001, **P < 0.01, *P < 0.05.

In this study, our objective was to simulate kidney injury *in vitro* and investigate the underlying molecular mechanisms involved. To achieve this goal, we utilized human renal TECs (HK-2) as an experimental model and use H_2_O_2_ as an inducer of oxidative stress to establish an injury model. H_2_O_2_ exposure induces HK-2 cells injury by increasing intracellular ROS production, decreasing cell viability, and increasing inflammatory marker expression (Park et al., 2017). Treatment of HK-2 cells with H_2_O_2_ induces oxidative stress, leading to cellular damage. By subjecting HK-2 cells to various concentrations of H_2_O_2_ and assessing cell viability through incubation (Figure 3A), we determined that a concentration of 300 μM H_2_O_2_ effectively induced a damage response in HK-2 cells. Once the appropriate stimulation concentration was established, we further evaluated changes in the glycolytic pathway. Treatment of HK-2 cells with 300 μM H_2_O_2_ resulted in a significant increase in HK gene expression levels (Figure 3B) and, consistent with this observation, concomitantly elevated intracellular HK enzyme activity (Figure 3C). Additionally, an increase in the pyruvate content was also observed (Figure 3D). These findings indicated that H_2_O_2_ stimulation promoted glycolysis in HK-2 cells, which is consistent with previous observations from mouse kidney injury studies. Subsequently, we conducted an analysis of the intracellular levels of MG (Figure 3E) and D-lactate (Figure 3F) and observed a significant elevation in their concentrations following the augmentation of glycolysis triggered by H_2_O_2_ stimulation. Intriguingly, our findings also revealed that H_2_O_2_ exerted minimal influence on the expression levels of GLO1 and GLO2 (Supplementary Figures S4A,B), whereas it exerted an inhibitory effect on the expression of LDHD (Supplementary Figure S4C). Immunofluorescence was then performed to detect MG-H1 expression, with or without the addition of semicarbazide (SC), which is a scavenger of MG. The observed decrease in MG-H1 expression following treatment confirmed the effectiveness of SC (Supplementary Figure S4D). The increase in MG after H_2_O_2_ stimulation may be significantly associated with increased glycolysis. Next, to assess the extent of LPO, flow cytometry analysis was performed on cells that were stimulated with H_2_O_2_, and the results revealed a significant increase in LPO levels (Figure 3H). Importantly, when SC was added, a notable reduction in LPO occurred, indicating the inhibitory effect of SC on LPO. In addition, we also assessed the intracellular levels of reduced GSH and observed a significant reduction in these levels in HK-2 cells H_2_O_2_ stimulation compared with those in normal cells (Figure 3G). However, upon the addition of SC, the GSH content was significantly restored, indicating that SC not only inhibited LPO but also protected cells from oxidative stress. Flow cytometry was subsequently used to detect the effects of a GLO1 inhibitor in H_2_O_2_-stimulated cells (Supplementary Figure S5). Our findings revealed that the presence of an MG inhibitor enhanced LPO. These results suggest that MG promotes GSH depletion and LPO. To further investigate the potential role of ferroptosis, an immunofluorescence technique was used to assess the intracellular 4HNE levels after H_2_O_2_ stimulation (Figure 3I). The results demonstrated a significant increase in 4HNE levels after H_2_O_2_ stimulation; however, this expression was reduced by the addition of SC. Additionally, we assessed the intracellular MDA contents (Figure 3J) and observed an increase in the MDA levels after H_2_O_2_ stimulation, whereas the addition of SC effectively mitigated this increase. These findings suggest a potential role of MG in facilitating ferroptosis. Collectively, our results demonstrate that glycolytic pathway activation, increased LPO, and changes in ferroptosis-related marker expression are closely associated with elevated MG levels in the H_2_O_2_-induced model of HK-2 cell injury. SC, which is a potent scavenger of methylglyoxaldehyde, inhibits LPO, restores intracellular GSH levels, and reduces oxidative stress-induced damage to cells, providing compelling evidence for the involvement of MG in cellular processes.

### MG facilitates the progression of ferroptosis in renal TECs

**FIGURE 4:**
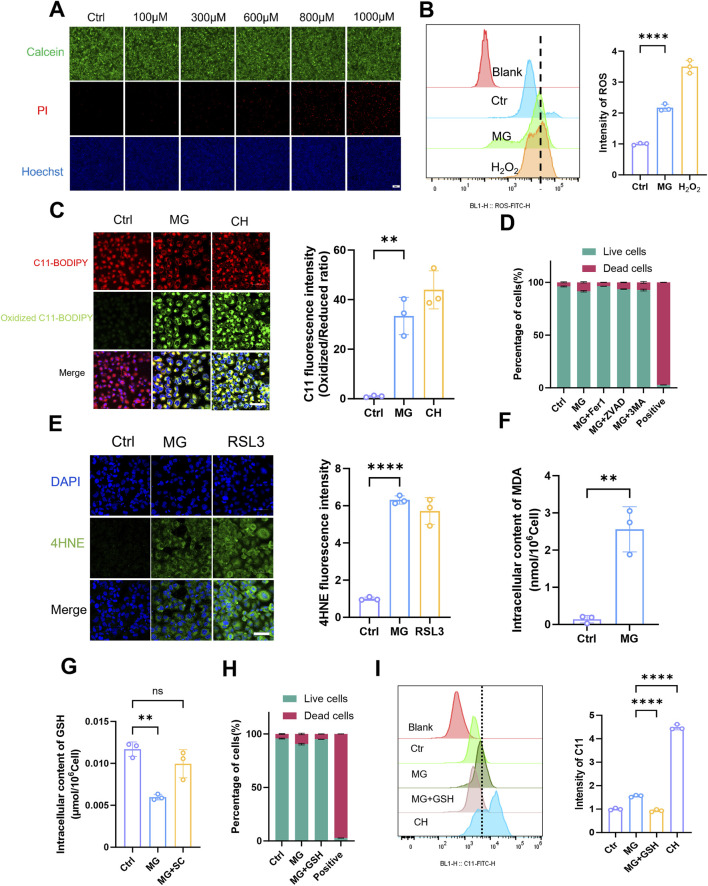
Methylglyoxal facilitates the advancement of ferroptosis in renal tubular epithelial cells. **(A)** Live/dead staining of HK-2 cells after incubated with varying concentrations of MG for 24 h. Scale bar: 20 μm (n = 3). **(B)** After HK-2 cells were stimulated with MG (600 μM) for 24h, the intracellular ROS was quantified by flow cytometry (n = 3). **(C)** After HK-2 cells were stimulated with MG (600 μM) for 24 h, intracellular lipid peroxidation was evaluated by fluorescence microscopy (left panel), and the relative quantitative analysis of fluorescence intensity (oxidized/reduced ratio) were also conducted (right panel). Scale bar: 20 μm (n = 3). **(D)** HK-2 cells were stimulated with MG for 24 h and then incubated with Fer-1 (5 μM), Z-vad (10 μM) and 3-MA (5 mM) for 1 h, respectively, before MG treatment. The live/dead cells ratio was detected by flow cytometry (n = 3). **(E)** After HK-2 cells were stimulated with MG (600 μM) for 24 h, immunofluorescence intensity of 4HNE was recorded by laser confocal microscopy (left panel) and the related quantitative analysis were also performed (right panel). Scale bar: 20 μm (n = 3) **(F)** HK-2 cells were stimulated with MG (600 μM) for 24 h, and the intracellular MDA content was measured (n = 3). **(G)** After HK-2 cells were stimulated with MG (600 μM) and SC (1 mM) for 24h, the intracellular GSH content was measured (n = 3). **(H)** HK-2 cells were stimulated with MG (600 μM) and GSH (1 mM) for 24 h and the live/dead cells ratio was detected by flow cytometry (n = 3) **(I)** The intracellular lipid peroxidation (C11) was detected by flow cytometry after HK-2 cells stimulated with MG and MG+GSH for 24 h (n = 3). ^****^ P < 0.0001, ^***^ P < 0.001, ^**^ P < 0.01, ^*^ P < 0.05.

After conducting extensive research on animal and cell injury models, we redirected our focus towards investigating the mechanism of MG itself. Building upon previous findings, we examined the toxic effects of exogenous MG on HK-2 cells by exposing them to various MG concentrations. The results from both the cell survival fluorescence assay (Figure 4A) and the CCK-8 assay (Supplementary Figure S6A) indicated that a concentration of 1 mM had the strongest toxic effect on HK-2 cells. The cytotoxic effect of MG on cells has been extensively investigated in numerous previous studies (Brouwers et al., 2014; Kim et al., 2020; Sekar et al., 2023; Kong et al., 2024). On the basis of a comprehensive analysis of both past and current research, we ultimately determined that the optimal concentration to achieve its effects on cells was 600 μM. After treatment with this MG concentration, intracellular ROS production in HK-2 cells was increased, and oxidative stress was induced to some extent (Figure 4B; Supplementary Figure S6B). Live-cell fluorescence imaging revealed that LPO levels were significantly increased in HK-2 cells that were stimulated with MG (Figure 4C), which was further confirmed through flow cytometry analysis (Supplementary Figure S6C); this result suggests a possible association between MG and ferroptosis. To elucidate the mode of cell death induced by MG, cells were treated with three inhibitors, and their effects on different modes of cell death were observed (Figure 4D). Among the ferroptosis, pyroptosis, and autophagy inhibitors that were tested, the ferroptosis inhibitor had the most significant protective effect against MG-induced damage in renal TECs; these results confirmed that MG primarily affects these types of cells via the ferroptotic pathway. Consistent with previous observations, as determined by an immunofluorescence assay (Figure 4E), the levels of 4-HNE in MG-treated HK-2 cells showed increased levels. Additionally, intracellular accumulation of ferrous ions was observed (Supplementary Figure S6D). These findings strongly suggest that MG can directly induce ferroptosis in renal TECs and that the intracellular content of MDA is also increased after treatment (Figure 4F). A decrease in GSH contents was observed both in the renal tissues of injured mice and in HK-2 cells in the injured state. Considering the interaction between the MG metabolic pathway and GSH, we observed a reduction in the intracellular GSH contents after cells were treated with MG and a significant recovery after the addition of the scavenger SC (Figure 4G); this result was further confirmed by a flow cytometry cell necrosis assay (Figure 4H). Stimulation of cells with MG as well as exogenous addition of GSH had inhibitory effects on cell death, suggestion that MG may promote ferroptosis by reducing GSH levels. On this basis, the induction of LPO by MG and the inhibitory effect of SC on LPO were detected by flow cytometry (Figure 4I); the results further suggested that MG may promote ferroptosis by reducing GSH levels. Taken together, these data suggest that MG may contribute to ferroptosis by affecting GSH regeneration and its ability to reduce oxidative stress.

### MG affects GSH metabolism by inhibiting the production of selenoproteins, thereby promoting ferroptosis in renal TECs

**FIGURE 5:**
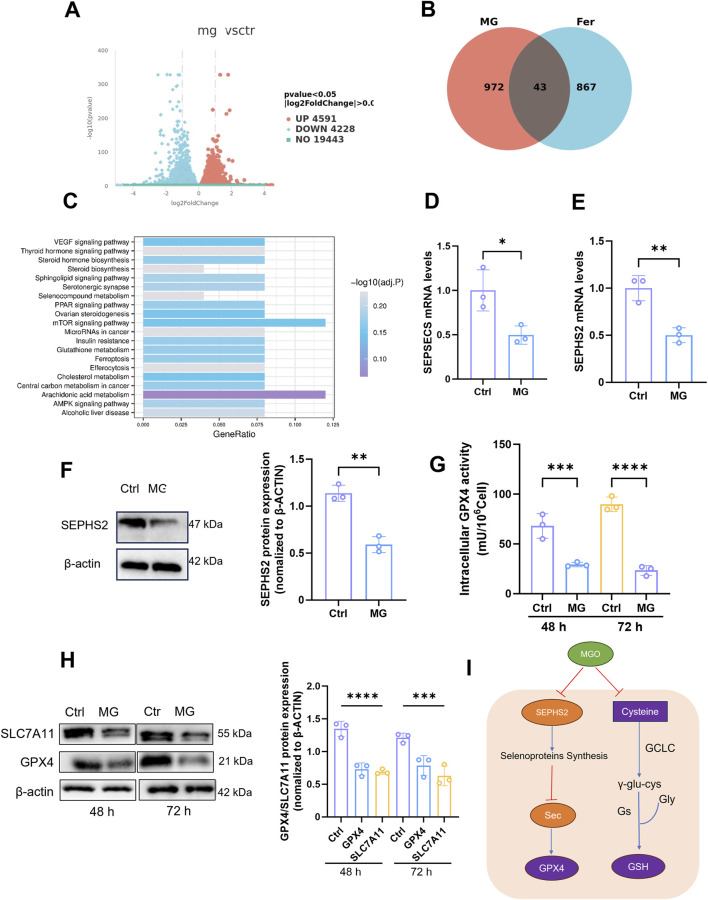
Methylglyoxal affects glutathione metabolism by inhibiting the production of selenoproteins, which leads to ferroptosis in renal tubular epithelial cells. **(A)** After HK-2 cells were stimulated with MG (600 μM) for 24h, RNA was extracted and genomic sequencing was performed (n = 3). The differentially expressed genes were obtained. **(B)** The differentially expressed genes were intersected with the existing ferroptosis-related gene set after conducting significant analysis and intersection genes (n = 3). **(C)** KEGG pathway analysis was performed on the existing differentially expressed genes (n = 3). **(D, E)** The relative gene expression levels of SEPSECS and SEPHS2 in HK-2 cells incubated with MG for 24 h. **(F)** SEPHS2 protein expression in HK-2 cells after incubated with MG for 24 h (left panel) and related quantification analysis were also performed (right panel). **(G)** GPX4 activity in HK-2 cells after incubated with MG (600 μM) for 48 h and 72 h. **(H)** Expression of GPX4 and SLC7A11 protein in HK-2 cells after incubation with MG for 48h and 72 h (left panel), the related quantification analysis was performed (right panel) (n = 3). **(I)** Schemic illustration of the impact of MG on Selenoprotein and GSH synthesis. ****P < 0.0001, ***P < 0.001, **P < 0.01, *P < 0.05.

On the basis of our previous studies, RNA-Seq was conducted on renal TECs that were incubated with exogenous MG to investigate changes in their gene expression profiles. Volcano plots (Figure 5A) and Venn diagrams (Figure 5B) were drawn to provide a comprehensive overview of the transcriptional changes induced by MG treatment in renal TECs. Volcano plots can identify significantly differentially expressed genes on the basis of their fold change and statistical significance, whereas the Venn diagrams reveal the overlap and uniqueness of these differentially expressed genes under different conditions, highlighting the potential commonalities and specificities of cellular responses to MG. To elucidate the molecular mechanisms underlying MG-induced ferroptosis and kidney injury, we performed an intersection analysis of these differentially expressed genes with known ferroptosis-related gene sets, thereby identifying targeted genes that may be associated with ferroptosis-related mechanisms. We subsequently performed gene set enrichment analysis (KEGG) on these intersecting genes and ultimately identified a metabolic pathway that was closely associated with GSH metabolism and selenide metabolism (Figure 5C). Selenium, which is an essential micronutrient, plays important roles in a variety of biological processes through its integration into selenocontaining proteins, namely, selenocysteines (Labunskyy et al., 2014). Selenocysteine is synthesized via specific pathways, and SEPHS2 is an enzyme that is indispensable for selenocysteine biosynthesis, especially for the formation of selenoproteins that perform critical functions, such as GPX4 (Burk and Hill, 2015). GPX4 is an important selenoprotein that prevents cells from undergoing ferroptosis, which is a regulated form of cell death that is induced by LPO (Yang et al., 2014). The production of GPX4 and other selenoproteins is highly dependent on SEPHS2, and SEPHS2 has been shown to be a potential target for cancer therapy because of its important role in cancer cell survival. Moreover, SEPHS2 functions beyond the synthesis of selenoproteins, as it is also required for the detoxification of selenides, which are toxic metabolites that are produced during selenocysteine biosynthesis (Carlisle et al., 2020). The SLC7A11 transporter contributes to selenium uptake and selenocysteine biosynthesis, which in turn affects the production of GPX4 and other selenoproteins that are indispensable for cellular REDOX homeostasis (Sato et al., 1999). The interaction between Se, GPX4, and GSH is essential for maintaining antioxidant defences and preventing the cellular damage caused by oxidative stress (Spallholz, 1994). After incubation with MG, we observed a significant decrease in the activity of key enzymes that are involved in selenoprotein production (Figures 5D,E) as well as a significant reduction in the expression of proteins that play key roles in the selenide metabolic pathway (Figure 5F), as measured at the genetic level. Specifically, both the enzyme activity and protein expression levels of GPX4, which has selenoprotein as its active centre, were significantly decreased (Figures 5G,H). Additionally, the expression of SLC7A11, which is associated with selenoprotein production, was also reduced (Figure 5H). These findings indicate that MG exerts a pronounced inhibitory effect on selenoprotein production. Furthermore, considering the strong connection between selenium compound metabolism and GSH synthesis, we investigated the impact of MG on GSH synthesis. Our results revealed that after incubation with MG, the expression of genes that are related to GSH synthesis was decreased. This included reduced activities of glutathione synthetase (GSS), Glutamate-Cysteine Ligase Catalytic (GCLC), and glutathione S-transferase (GSTS) (Supplementary Figures S7A–C). Overall, our findings suggest that MG binds to GSH, inhibits GSH resynthesis, and affects crucial enzyme activities that are involved in GSH synthesis and GSH-related selenoprotein generation, thereby promoting ferroptosis (Figure 5I).

## Discussion

MG, which is a highly reactive dicarbonyl compound, may be recognized as an important contributor to the development of kidney injury. In addition, previous studies have shown that MG and D-lactic acid levels are significantly increased in aristolochic acid-induced and STZ-injected mouse models of kidney injury (Lin et al., 2011; Lin et al., 2021), and these levels are significantly correlated with kidney injury. Our study demonstrated that MG levels are significantly increased in a mouse model of folate-induced kidney injury; these results highlight the potential role of MG in the progression of kidney injury. The observed increase in MG concentrations in the context of acute and chronic kidney injury suggests a close association between MG accumulation and renal impairment. While our animal data establish correlative relationships, the causal role of MG requires further *in vivo* validation through targeted interventions It has been shown that MG is derived mainly from glycolysis (Luengo et al., 2019; Kong et al., 2024). Under physiological conditions, proximal convoluted tubule cells produce ATP primarily through the oxidative phosphorylation of acetyl-CoA from fatty acid oxidation (FAO), and these cells are barely able to use glucose as an energy substrate (Bataille et al., 2018; Lakhia et al., 2018). This phenomenon is attributed to the expression of key enzymes of the glycolytic pathway (hexokinase, fructose-6-phosphate activating enzyme, and pyruvate kinase), which are not normally present in the proximal tubules (Guder and Ross, 1994; Lee et al., 2015). This process is also dependent on aerobic metabolism, which makes it more susceptible to oxidative stress damage (Daenen et al., 2018; Flemming et al., 2018). However, during the progression of renal injury, energy metabolism changes, and proximal renal TECs are unable to support FAO because of the high oxygen demand after injury; thus, the mechanism for supplying energy changes to glycolysis (Legouis et al., 2022), which provides the conditions for MG production. We demonstrated that this occurred through increased hexokinase activity and elevated pyruvate contents in the renal tissue of injured mice. Unlike classical AKI models showing ROS-mediated pyruvate depletion (Legouis et al., 2022), our folate-injury model revealed elevated pyruvate, likely due to glycolytic surge overwhelming catabolism and MG-induced PDH/LDH inhibition. Future ^13^C-pyruvate tracing will delineate its fate in MG-rich microenvironments. In addition, the activities of key enzymes that are involved in MG metabolism, including GlO1, GlO2, and D-lactate dehydrogenase, were significantly reduced in the kidney injury model, indicating a decreased MG detoxification capacity. Under pathological conditions, the double injury of increased MG production and decreased MG degradation in renal tissues leads to the accumulation of this toxic metabolite, which may further exacerbate renal injury.

Our investigation of the mechanisms by which MG affects renal TECs revealed significant cytotoxic effects associated with MG exposure. In HK-2 cells that were treated with different concentrations of MG, a cell viability assay revealed that MG reduced cell viability in a dose-dependent manner, indicating that MG exerts a direct cytotoxic effect on these cells. Although the concentration in the human body may not reach the concentration used for exogenous MG treatment, some studies have shown its toxic effect on cells (Sena et al., 2012; Shopit et al., 2020). The critical role of renal TECs in renal function and their high energy requirements have been previously described, and these findings are particularly relevant. The shift to glycolysis that was observed under pathological conditions in our study is consistent with the metabolic reprogramming that is known to occur during kidney injury, suggesting that the effect of MG on these cells may be a key factor in the progression of kidney injury.

A significant decrease in the reduced GSH content was observed in the renal tissues of mice with renal injury, indicating a reduction in antioxidant capacity. This finding is further supported by decreased expression of glutathione peroxidase and cystine/glutamate antiporter, as well as increased levels of MDA, suggesting the occurrence of LPO in injured kidney tissues. Immunohistochemistry and immunofluorescence techniques confirmed the upregulation of 4HNE, which is a specific end product of ferroptosis, further confirming its involvement in kidney injury, as previously demonstrated (Ide et al., 2021; Wang et al., 2021). *In vitro* experiments also revealed increased LPO in cells that were treated with H_2_O_2_ followed by a Glo1 inhibitor. These renal injuries were accompanied by an increase in MG, suggesting its potential role in promoting the initiation and progression of ferroptosis in injured renal tissues. However, our study has limitations, as the cell damage caused by H_2_O_2_ may not fully mimic the complex pathological conditions observed in humans, which requires further exploration.

The direct promotion of ferroptosis in renal TECs by MG was investigated to evaluate its role in this form of cell death. Our findings revealed a significant increase in intracellular LPO after MG treatment, as confirmed by flow cytometry analysis and live-cell imaging. This increased LPO, which is a hallmark of ferroptosis, suggests a direct association between MG exposure and the induction of this specific type of cell death. This study elucidates the pivotal role of MG in bridging oxidative stress and ferroptosis during kidney injury. As demonstrated in Figure 3, hydrogen peroxide-induced oxidative stress markedly elevated MG levels through glycolytic activation, which subsequently amplified lipid peroxidation and depleted glutathione reserves. The reversal of these effects by the MG scavenger SC confirmed MG’s central causative role. Exogenous MG exposure (Figure 4) further revealed two distinct ferroptosis-promoting mechanisms: covalent binding-mediated glutathione depletion, which crippled glutathione peroxidase activity, and suppression of selenoprotein biosynthesis, evidenced by diminished GPX4 and SLC7A11 expression (Figure 5). The pronounced protective effect of the ferroptosis inhibitor Fer-1—compared to apoptosis or autophagy inhibitors—definitively established ferroptosis as the predominant cell death pathway. These findings position MG as a metabolic linchpin in renal injury: glycolytic reprogramming generates excess MG, which perpetuates tubular cell death by disrupting antioxidant defenses and driving iron-dependent lipid peroxidation, thereby creating a self-reinforcing pathological cycle. Furthermore, the levels of 4HNE, which is the specific end product associated with ferroptosis, was elevated in HK-2 cells that were exposed to MG, providing additional evidence for the proferroptotic effect of MG. Notably, when different forms of cell death were inhibited with specific inhibitors, ferroptosis inhibitors had the strongest protective effect on MG-treated cells. These findings further confirmed that MG primarily affects renal TECs through activation of the ferroptotic pathway, thereby exacerbating renal injury. However, it should be noted that our study has certain limitations; for example, owing to its *in vitro* nature, it may not fully capture the complex interactions among various factors that occur within an *in vivo* environment. Additionally, although our study revealed the proferroptotic role of MG, further research is needed to elucidate the specific intracellular signalling cascades and molecular interactions that mediate MG-induced ferroptosis.

In terms of the inhibitory effect of MG on selenoproteins and its impact on GSH metabolism, our study demonstrated that MG not only forms a complex with GSH but also significantly hinders the biosynthesis of selenoproteins, which serve as crucial components of antioxidant defence mechanisms and play active roles in the glutathione peroxidase GPX4. RNA-Seq analysis of renal TECs that were treated with exogenous MG revealed a decrease in the expression of genes that are involved in selenoprotein synthesis. This observation was further supported by a noticeable decrease in the expression level of selenophosphate synthetase, thus indicating a clear inhibitory effect of MG on selenoprotein production. Selenium compounds play pivotal roles in the synthesis of key antioxidants, such as glutathione peroxidase. Our findings suggest that the MG-mediated inhibition of selenoprotein production may contribute to the observed oxidative stress and ferroptosis in renal injury. Additionally, we observed decreased expression levels of genes involved in GSH synthesis, including the reduced activity of glutathione synthetase (GSS), Glutamate-Cysteine Ligase Catalytic (GCLC), and glutathione S-transferase (GSTS), after MG treatment. This suggests that the effects of MG extend beyond binding to GSH and involve the inhibition of GSH resynthesis and enzymes that play key roles in its synthesis, thereby promoting ferroptosis.

## Conclusion

**FIGURE 6:**
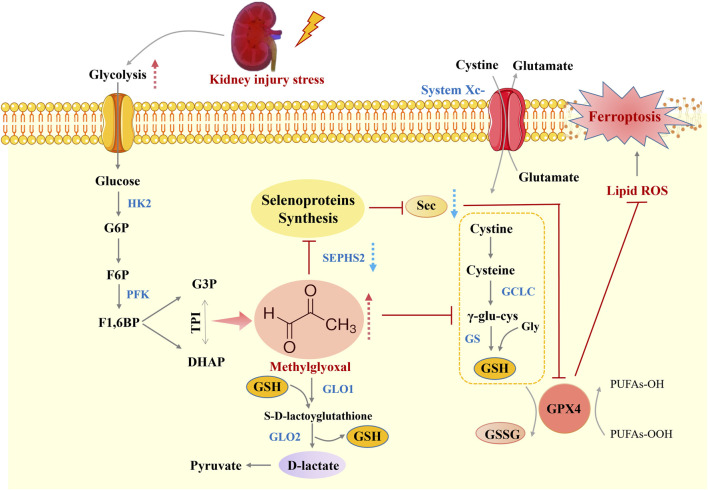
Schematic overview of MG-induced ferroptosis during kidney injury. The Figure depicts the mechanistic link between methylglyoxal accumulation and ferroptosis in renal tubular epithelial cells during kidney injury. MG, a byproduct of heightened glycolysis, promotes oxidative stress and impairs selenoprotein synthesis, which is crucial for antioxidant defence. This leads to glutathione depletion, lipid peroxidation, and ultimately ferroptosis, thus exacerbating kidney injury. This figure was designed by the authors.

Taken together, our findings establish the key role of MG in the pathogenesis of kidney injury, highlighting how kidney injury leads to increased MG levels, which in turn promote ferroptosis and exacerbate kidney injury (Figure 6). We observed elevated MG levels in both clinical and experimental models of kidney injury and linked their accumulation to a metabolic shift towards glycolysis and renal impairment. The direct cytotoxic effects on renal TECs, promotion of oxidative stress, and association with ferroptosis highlight the deleterious role of MG in exacerbating renal injury. Furthermore, the inhibition of selenoproteins by MG and its effect on GSH metabolism provide a potential molecular mechanism for its ferroptotic activity, which is a key factor in the progression of kidney injury. Although our study provides valuable insights, it is limited by the use of *in vitro* experiments and needs to be further validated by *in vivo* studies. Future studies should aim to elucidate the detailed signalling pathways involved and evaluate potential therapeutic interventions to target MG-mediated effects. This study provides a solid foundation for understanding the role of MG in renal injury and ferroptosis and provides new directions for the development of novel therapeutic strategies.

## Data Availability

The datasets presented in this study can be found in online repositories. The names of the repository/repositories and accession number(s) can be found in the article/Supplementary Material.
