# Supplementary material for: Methylglyoxal induces oxidative stress and ferroptosis of renal tubular epithelial cells in acute and chronic kidney injury mice
Source: Front Cell Dev Biol. 2025 Sep 19;13:1604575. doi: 10.3389/fcell.2025.1604575 (PMC12492953; doi:10.3389/fcell.2025.1604575)
Supplement: Supplementary file 1 [file DataSheet1.docx]

**Supplementary Material**

**Methylglyoxal exacerbates kidney injury by promoting the ferroptosis of renal tubular epithelial cells**

Yongzheng Zhang^1, 2,^ , Xuwu Zhang^1, 2^, Jiayu Ren^2, 3^ , Hao Sun^2, 4,^ *, Zhe Yang ^1,*^,Jianning Wang^1,^ *

^1,^ Shandong First Medical University & Shandong Academy of Medical Sciences, Department of Urology, The First Affiliated Hospital of Shandong First Medical University, Jinan 250014, China;

^2,^ Medical Science and Technology Innovation Center, Shandong First Medical University & Shandong Academy of Medical Sciences, Jinan 250117, China;

^3,^ School of Public Health, Shandong First Medical University & Shandong Academy of Medical Sciences, Jinan 250117, China.

^4,^ School of Preventive Medicine Sciences (Institute of Radiation Medicine), Shandong First Medical University & Shandong Academy of Medical Sciences, Jinan 250117, China.

* Correspondence:

Hao Sun, [sunhaofs@163.com](mailto:sunhaofs@163.com)

Zhe Yang, yangz910306@163.com

Jianning Wang, [docjianningwang@163.com](mailto:docjianningwang@163.com)

**Running head:** Methylglyoxal accumulation worsens kidney injury.

**
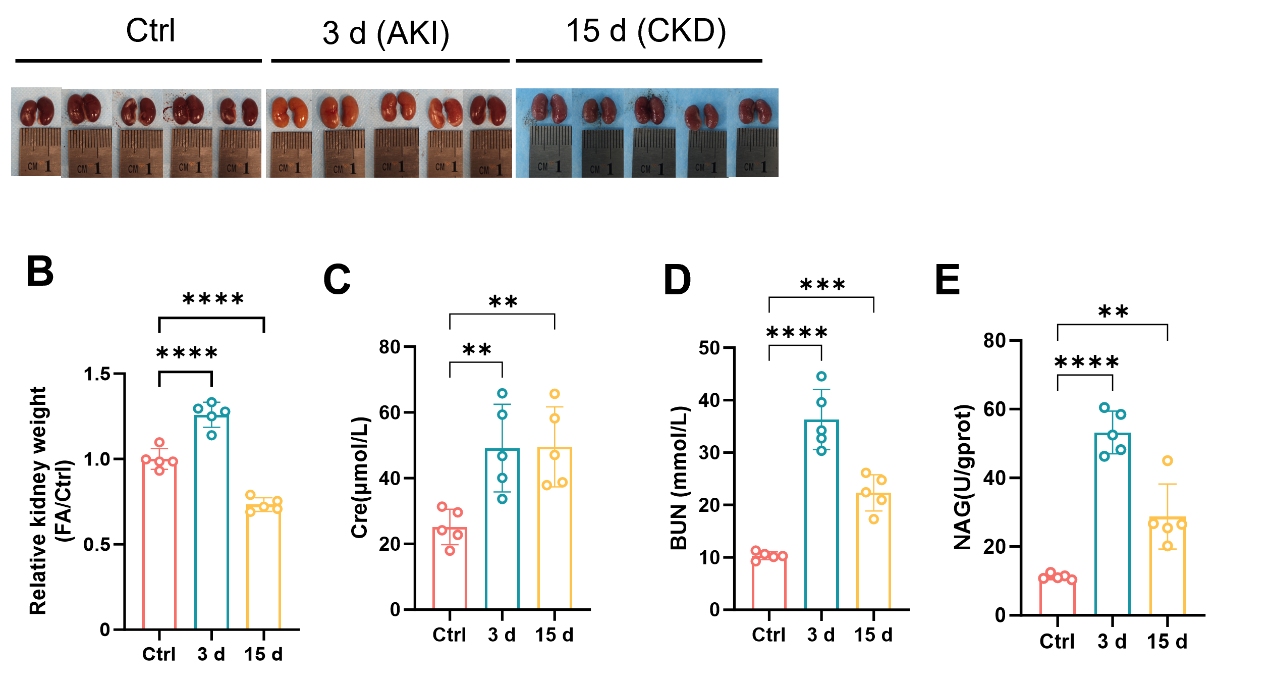
**

**Supplementary Figure S1.** (A) Morphology of kidneys in mice injected with folic acid at different stages (n=5). (B) Kidney weight of mice after injected with folic acid at different stages (n=5). (C-D) Blood creatinine and Urea nitrogen levels in mice at different stages after folic acid injection (n=5). (E) N-acetyl-β-D-glucosaminidase content in kidney tissues of mice at different stages after folic acid injection (n=5). ****P< 0.0001, ***P < 0.001, **P < 0.01, *P < 0.05.

**
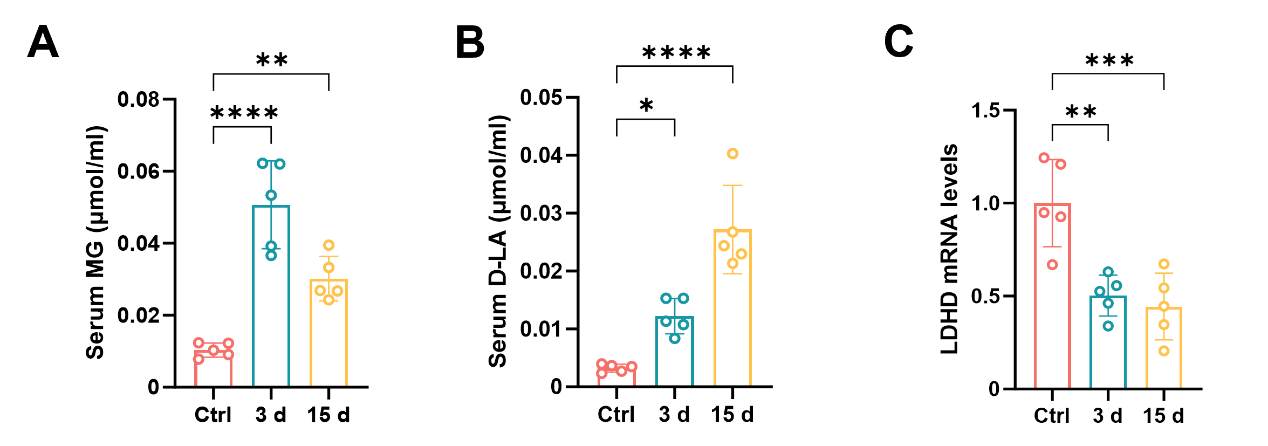
**

**Supplementary Figure S2.** (A-B) The content of MGO and D-LA in mice serum at different stages after folic acid injection (n=5). (C) Expression of the LDHD gene in kidney tissue of mice during various stages of folic acid injection (n=5). ****P< 0.0001, ***P < 0.001, **P < 0.01, *P < 0.05.

**
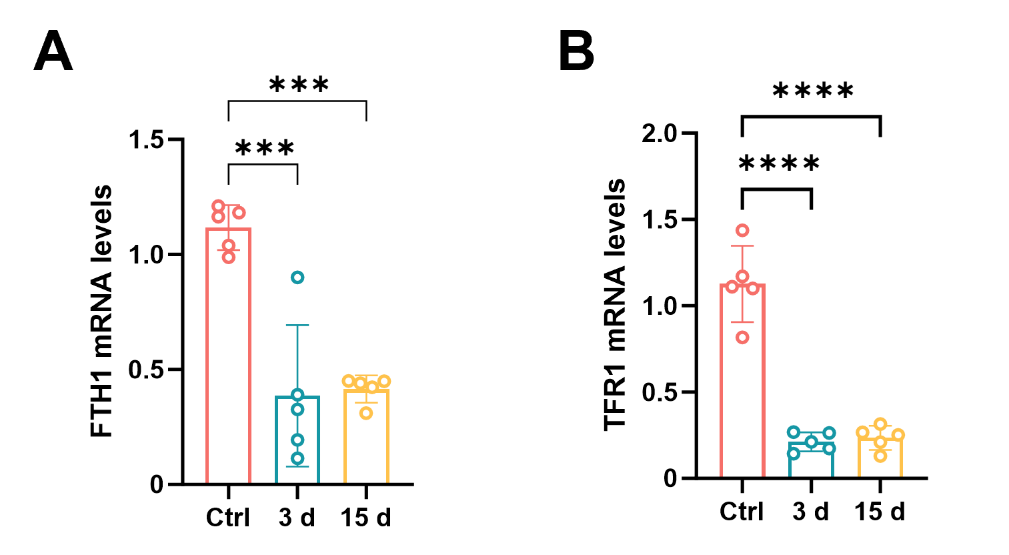
**

**Supplementary Figure S3.** (A-B) The relative gene expression of FTH1 and TFR1 in kidney tissue of mice during various stages of folic acid injection(n=5). ****P< 0.0001, ***P < 0.001, **P < 0.01, *P < 0.05.

**
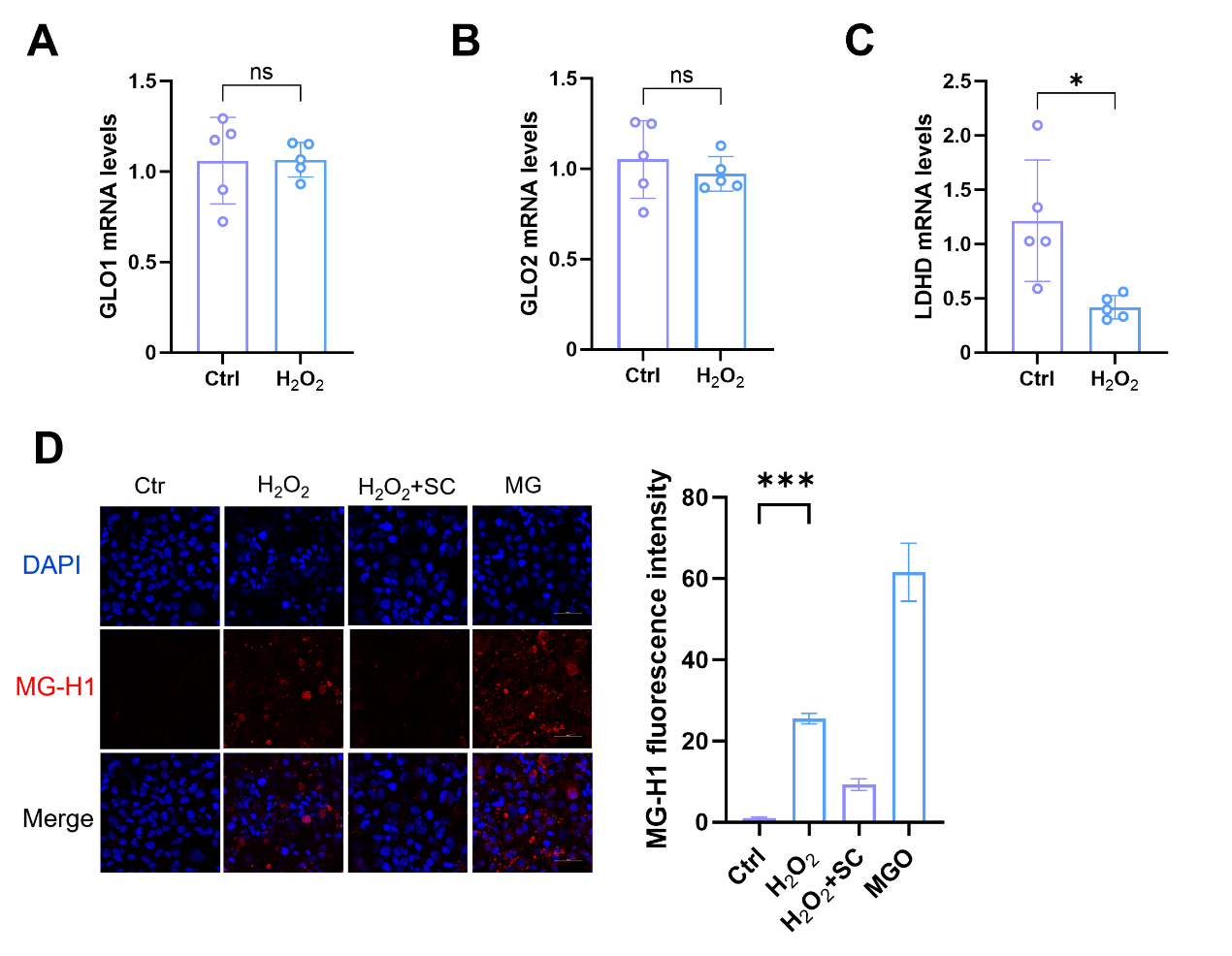
**

**Supplementary Figure S4.** (A-C) The relative gene expression of GLO1, GLO2, and LDHD in HK-2 cells after stimulated by H_2_O_2_ (n=3). (D) The immunofluorescence images of MG-H1 in HK-2 cells after stimulated by H_2_O_2_, H_2_O_2_+SC, and MGO, respectively (n=3). Scale bar: 20 μm. ****P< 0.0001, ***P < 0.001, **P < 0.01, *P < 0.05.

**
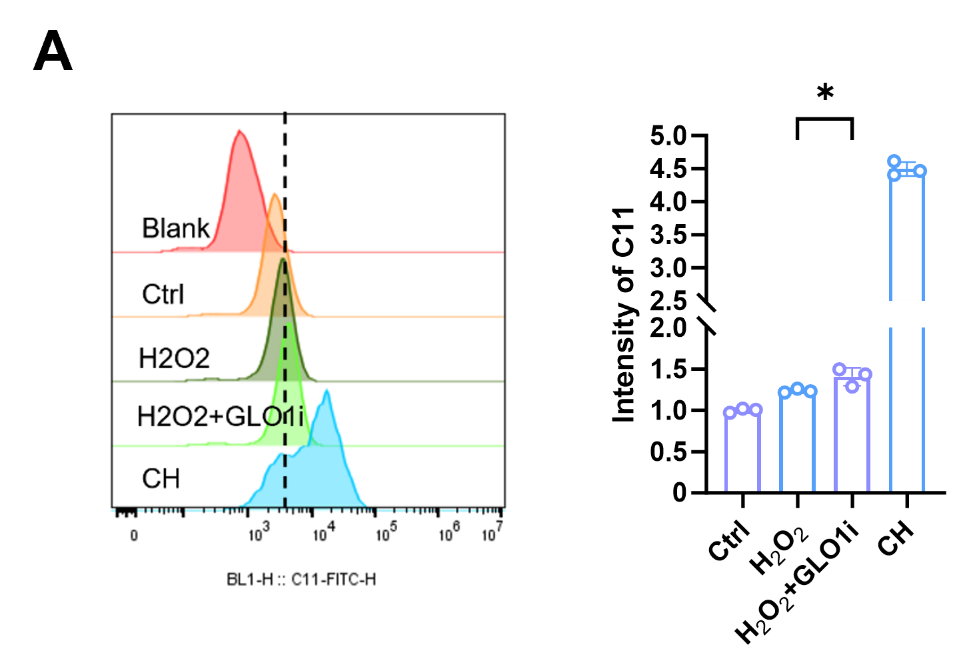
**

**Supplementary Figure S5.** Flow cytometry was employed to assess the intensity of lipid peroxidation in HK-2 cells upon stimulation with H_2_O_2_ and subsequent addition of GLO1 inhibitor (n=3). ****P< 0.0001, ***P < 0.001, **P < 0.01, *P < 0.05.

**
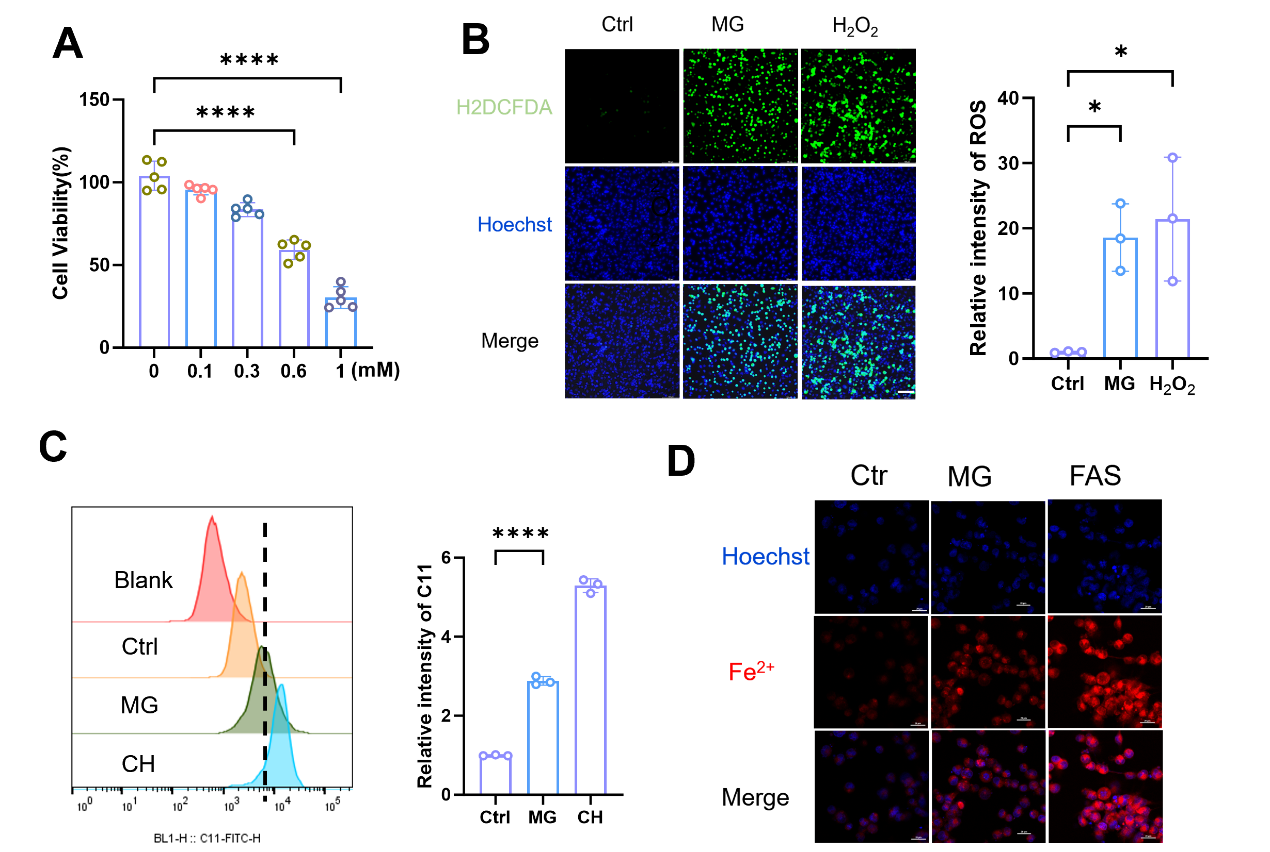
**

**Supplementary Fig. S6.** (A) Cell viability of HK-2 cells was assessed by CCK-8 assay after incubation with MGO (n=3). (B) Detection of ROS fluorescence in HK-2 cells following incubation with MGO (n=3). (C) Assessment of lipid peroxidation in HK-2 cells following incubation with MGO using flow cytometry (n=3). (D) After incubating HK-2 cells with MGO, the fluorescence of Fe^2+^ was detected by fluorescence microscopy, with FAS serving as the positive control for ammonium ferrous sulfate (n=3). ****P< 0.0001, ***P < 0.001, **P < 0.01, *P < 0.05.

**
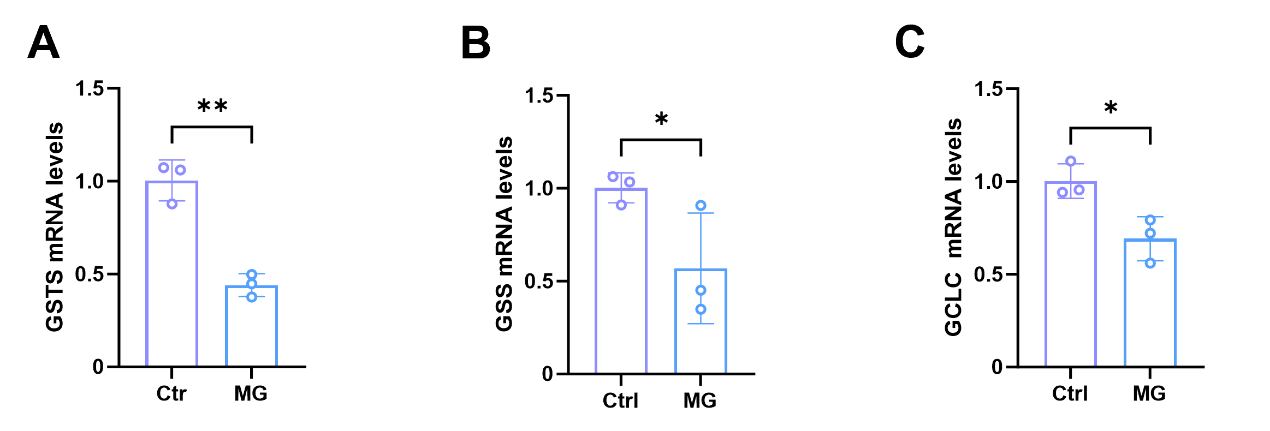
**

**Supplementary Figure S7.** (A-C) The relative gene expression level of GSTS, GSS, and GCLC in HK-2 cells following incubation with MGO (n=3). ****P< 0.0001, ***P < 0.001, **P < 0.01, *P < 0.05.

**Table**

| Gene | Species | Forward primer | Reverse primer |
| --- | --- | --- | --- |
| Glo1 | Mouse | TGGATTTGGTCACATTGGGATTGC | GGTCTTGAATGAACGCCAGTCC |
| Glo2 | Mouse | GGCTGAAGGTTTATGGAGGTGATG | CGAAGTATGGCAGGGTGTTGAC |
| Hexokinase | Mouse | TGATCGCCTGCTTATTCACGG | AACCGCCTAGAAATCTCCAGA |
| Ldhd | Mouse | GTGTCAACCTCCTGATGCTGTG | CGGTGCCTGTGCCAAATGG |
| Gpx4 | Mouse | ATAAGAACGGCTGCGTGGTGAAG | TAGAGATAGCACGGCAGGTCCTTC |
| Slc7a11 | Mouse | ACCACCATCAGTGCGGAGGAG | ATGGAGCCGAAGCAGGAGAGG |
| Tfr | Mouse | GGCTCTGGCTCTCACACTCTC | GGCATTTGCGACTCCCTGAATAG |
| Fth1 | Mouse | TGCCAAATACTTTCTCCACCAATCTC | CCCGCTCTCCCAGTCATCAC |
| Ptgs2 | Mouse | GTGCCTGGTCTGATGATGTATGC | TGAGTCTGCTGGTTTGGAATAGTTG |
| Acsl4 | Mouse | GCGTTCCTCCAAGTAGACCAACC | ACGTTCACACTGGCCTGTCATTC |
| β-actin | Mouse | GGCTGTATTCCCCTCCATCG | CCAGTTGGTAACAATGCCATGT |
| GLO1 | Human | TTCGGTCATATTGGAATTGCTGTTC | GCCATCAGGATCTTGAATAAATGCC |
| GLO2 | Human | AGATCACTCACCTGTCCACACTG | CGGGCTTGCTCACGAAGTAAC |
| Hexokinase | Human | CGTGCCCGCCAGAAGACATTAG | CTTGCTCAGACCTCGCTCCATTTC |
| LDHD | Human | TGATGACGCCGAGGAACTGG | GCTGCCGCTTGCCCATTC |
| GPX4 | Human | CCCGATACGCTGAGTGTGGTTTG | TCTTCGTTACTCCCTGGCTCCTG |
| SLC7A11 | Human | ACGGTGGTGTGTTTGCTGTCTC | GCTGGTAGAGGAGTGTGCTTGC |
| TFR1 | Human | CGGCAAGTAGATGGCGATAACAG | CACAGCAATAGTCCCATAGCAGATAC |
| FTH1 | Human | TGAGCAGGTGAAAGCCATCAAAG | GAGATATTCCGCCAAGCCAGATTC |
| PTGS2 | Human | TCCACCAACTTACAATGCTGACTATG | ATCATCAGGCACAGGAGGAAGG |
| ACSL4 | Human | GCTCTGTCACACACTTCGACTCAC | TTCCCTGGTCCCAAGGCTGTC |
| β-ACTIN | Human | GATCATTGCTCCTCCTGAGC | ACTCCTGCTTGCTGATCCAC |

**Table S1. The list of primer sequences utilized in this study.**
